# Fuzzy Evaluation of Pharmacokinetic Models

**DOI:** 10.1155/2018/1983897

**Published:** 2018-11-01

**Authors:** Carlos Sepúlveda, Oscar Montiel, José M. Cornejo Bravo, Roberto Sepúlveda

**Affiliations:** ^1^Instituto Politécnico Nacional, Centro de Investigación y Desarrollo de Tecnología Digital (CITEDI-IPN), Av. Instituto Politécnico Nacional, No. 1310, Col. Nueva Tijuana, 22435 Tijuana, BC, Mexico; ^2^Facultad de Ciencias Químicas e Ingeniería, Universidad Autónoma de Baja California, Calzada Universidad 14418, Parque Industrial Internacional Tijuana, 22390 Tijuana, BC, Mexico

## Abstract

Population pharmacokinetic (PopPK) models allow researchers to predict and analyze drug behavior in a population of individuals and to quantify the different sources of variability among these individuals. In the development of PopPK models, the most frequently used method is the nonlinear mixed effect model (NLME). However, once the PopPK model has been developed, it is necessary to determine if the selected model is the best one of the developed models during the population pharmacokinetic study, and this sometimes becomes a multiple criteria decision making (MCDM) problem, and frequently, researchers use statistical evaluation criteria to choose the final PopPK model. The used evaluation criteria mentioned above entail big problems since the selection of the best model becomes susceptible to the human error mainly by misinterpretation of the results. To solve the previous problems, we introduce the development of a software robot that can automate the task of selecting the best PopPK model considering the knowledge of human expertise. The software robot is a fuzzy expert system that provides a method to systematically perform evaluations on a set of candidate PopPK models of commonly used statistical criteria. The presented results strengthen our hypothesis that the software robot can be successfully used to evaluate PopPK models ensuring the selection of the best PopPK model.

## 1. Introduction

**Figure 1:**
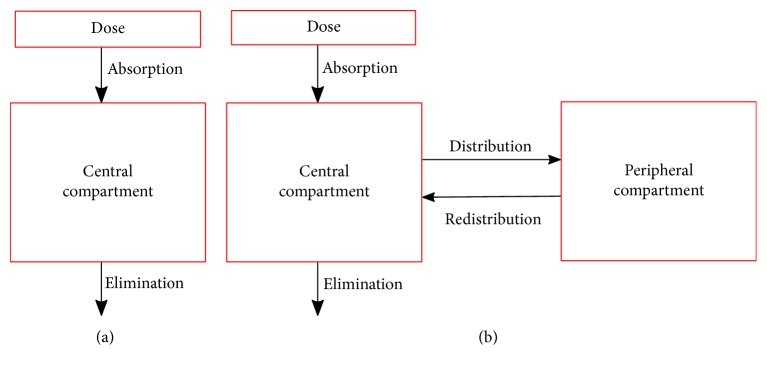
Example of the body viewed as (a) one-compartment model or (b) two-compartment model. The central compartment represents the plasma and tissues, and the peripheral compartment represents those tissues that take up the drug at a slower rate.

Pharmacokinetics (PK) is a subdivision of pharmacology in which mathematical models are developed to study the processes of absorption, distribution, and elimination of a drug once a given dose has been administered to a given individual [1]. These mathematical models are derived from representing the body as a system of compartments [2] (Figure 1), where the transfer rates of absorption, distribution, redistribution, and elimination between these compartments can be used to determine the parameters of the PK model such as clearance (Cl) and volume of distribution (*V*) that allow us to predict the drug concentration in plasma (*C*_p_) in a given time [3].

Let us consider the one compartment PK model of Figure 1, where the rate of elimination of the drug presented in the body decreases in proportion to the *C*_p_, that is, with a first-order elimination process *k*_e_ and the drug administered as a single intravenous bolus dose (*D*); the kinetic of *C*_p_ in the compartment—the body—at time *t* > 0 is depicted by the following deterministic differential equation:

(1) $$\begin{array}{c} \frac{dC_{{\text{p}}_{t}}}{dt}=-k_{\text{e}}C_{{\text{p}}_{t}},\\ C_{{\text{p}}_{0}}=\frac{D}{V}. \end{array}$$

In this case, the term *k*_e_ is given by

(2) $$\begin{array}{c} k_{\text{e}}=\frac{\text{Cl}}{V}, \end{array}$$

where *V* is the distribution volume of the drug within the body and Cl measures the ability of the liver and the kidney, mainly to extract a drug from the body. In this context, Cl and *V* are fixed parameters that describe the drug concentration of a given dose *D* over time *t*. Equation (1) has the following explicit solution:

(3) $$\begin{array}{c} C_{{\text{p}}_{t}}=\frac{D}{V}e^{-\left(\text{Cl}/V\right)\cdot t}. \end{array}$$

The model (3) states a relationship between the independent variable *t* and the dependent variable *C*_*p*_ [1, 4].

PopPK models are designed to analyze drug behavior in a group of individuals; hence, it is possible to generalize the model for similar individuals that were not subject of the study. NLME modeling is the main current approach for PopPK model development; it allows estimation of parameters in the presence of different levels of variability, and it is commonly used when it is not possible to obtain the complete information on repeated measurements of individuals [2]. Most of the time, the process to find the final PopPK model that better represents the data behavior requires the development of several PopPK models. It is common to use interchangeable software programs like R or SAS to apply various evaluation criteria to select which PopPK model is “better” among all the possible PopPK models [5–7]. These evaluation criteria range from graphical analysis to statistical methods [8]. The graphical analysis is limited to expert modelers, and besides that, it can be time-consuming and is subject to human error or misinterpretation.

The statistical methods such as the estimated error variance (MSE), the objective function value (OFV), the Akaike's information criterion (AIC), or the Bayesian information criterion (BIC), also known as Schwarz criterion (CS) offer quantitative terms (values) which are much easier to interpret and for consequence more easily to exclude or accept. However, the respective values may be contrasted between PopPK models when more than one statistical criterion is used complicating in this way ensuring the selection of the best PopPK model, thus becoming a multiple criteria decision making (MCDM) problem [9].

Fuzzy expert systems is an area where artificial intelligence and fuzzy logic meet to make robots backing or even replace human experts that want to do automated tasks [10]. The fuzzy logic has been recognized as an essential problem-solving technique when human evaluations are required for interpretation of medical findings [11], as well as in problems where is necessary for managing data with uncertainty such as [12], where a type-2 fuzzy logic methodology is used to help a neural network to handle complex time series data.

We contribute with a new software robot to facilitate the selection of the best pharmacokinetic model by an automated process when the comparison of the implemented evaluation criteria values among the models cannot be categorically determined, addressing the problem as a decision problem. We present results using a software robot to select the best PopPK model of tobramycin by an automated process.

The organization of this work is as follows: in Section 2, an overview of nonlinear mixed-effects modeling is provided. In Section 3, the implementation of the software robot is explained. In Section 4, the parameter estimation results and the outcomes of the software robot are presented. In Section 5, an analysis of the results is exposed. Finally, in Section 6, the conclusions are given.

## 2. Nonlinear Mixed-Effects Model Framework

In mixed-effects models, the data set is longitudinal, i.e., it is composed of repeated samples of the same individuals [13, 14]. This is the case of the PopPK experimental data; it consists of a vector *y*_*ij*_ of samples of the *j* − th measurement (where, *j*=1,…, *n*) of *C*_*p*_ in the *i* − th individual (where, *i*=1,…, *n*). Therefore, we can obtain the sample *y*_*ij*_ for the individual *i* at the time *t*_*ij*_ by using the model:

(4) $$\begin{array}{c} y_{ij}=f\left(x_{ij},\phi_{i}\right)+\varepsilon_{ij};\;\varepsilon_{ij}∼\left(0,\sigma^{2}\right), \end{array}$$

where *f*(·) is a nonlinear function as Equation (3) relating a vector of known values *x*_*ij*_ (e.g., dose and time) to the unknown parameter vector *ϕ*_*i*_(e.g., (Cl_*i*_, *V*_*i*_)^T^) and the variable *ε*_*ij*_ in the second term is the residual error that is assumed to be normally distributed with mean zero and variance *σ*^2^. It is sensible to expect that each individual has a different parameter vector *ϕ*_*i*_, and this is described in a second stage by the covariate model:

(5) $$\begin{array}{c} \phi_{i}=g\left(z_{i};\theta \right)+\eta_{i};\;\eta_{i}∼\left(0,\omega^{2}\right). \end{array}$$

Equation (5) describes the variation among different individuals accounted through the individual-specific parameters *ϕ*_*i*_.

The function *g*(·) relates the specific covariates *z*_*i*_ of the individuals to the population parameters *θ*. *η*_*i*_ is a (*q* × 1) vector containing the random effects that have a normal distribution with a covariance matrix *ω*^2^; other distributions for random effects exist [14].

### 2.1. Estimation of Population Parameters

Since the PopPK data are longitudinal, we can assume that the individuals are sampled randomly from the population, and therefore it is practical to assume that the *η*_*i*_'s are also randomly sampled (even though they are not observable) to conduct the estimations of the parameters of (4) and (5) by optimizing the likelihood function defined by the following equation:

(6) $$\begin{array}{c} L\left(\theta ,\Omega ,\Sigma \left|y\right.\right)\;≜\;p\left(y\left|\beta ,\Omega ,\Sigma \right.\right), \end{array}$$

where *L*(·) is the likelihood function, Ω is the variance-covariance matrix of all the *η*_*i*_'s, and Σ the residual variance-covariance matrix of all the *ε*_*i*_'s where *p*(**y**|*β*, Σ, *η*) is the conditional probability density of all measurements [15]. If *p*(**y**|*β*, Σ, *η*) is explicit, the likelihood function *L*(·) is explicit too, and the exact maximum likelihood estimators can be applied [16], otherwise the estimation of model parameters is made using linear approximations for (6) [17]. Either way, using an explicit likelihood function or an approximation, the precision in the estimated vector $\hat{\theta }$ depends not only in the residual variability but also in the estimated variance-covariance matrix $\hat{\Omega }$, which is calculated using the inverse Hessian matrix of the −log *L*(·).

### 2.2. Nonlinear Mixed-Effects Model Evaluation Criteria

In the literature referring on the analysis and development of NLME models and PopPK models, the researchers frequently highlighted three statistical criteria to carry out the different evaluations of the model and thus decide which PopPK model they should select within a collection of PopPK models they are considering.

The first two evaluation criteria that we are going to present in this work are the Akaike information criterion (ACI) [18] and the Schwarz's information criterion (SC) [19]. These evaluation criteria are derived from information theory and a Bayesian approach and are commonly used for evaluating nonlinear mixed-effects models when the parameters of the model were estimated by the maximum likelihood method. The AIC criterion provides a balance between the goodness of the fit of the model and the number of parameters required to obtain the fit [20] as is shown in the following equation:

(7) $$\begin{array}{c} \text{AIC}=N\cdot log\left(\text{OFV}\right)+2\cdot \text{NP,} \end{array}$$

where *N* is the number of observations and NP the number of parameters. The final and optimal model is the one with the smallest AIC value. Unlike the AIC criterion, the *SC* criterion accepts equal probability for each model and for every possible parameter value under the model as is shown in the following equation:

(8) $$\begin{array}{c} SC=N\cdot log\left(\text{OFV}\right)+\text{NP}\cdot log\text{ NP.} \end{array}$$

The third evaluation criterion is the estimated error variance also known as the mean square error (MSE). This is based on the simple variance estimator shown in the following equation:

(9) $$\begin{array}{c} {\hat{\sigma }}_{i}^{2}=\frac{{\sum^{}}_{i=1}^{m}y_{i}-\hat{y}}{n}. \end{array}$$

The use of the MSE is due to the residuals, and it also contains essential information on the quality of the model. Specifically, the estimated variance in the responses, where *y* is a vector of observed values and ${\hat{y}}_{i}$, represents a vector of *n* predictions.

The version of Equation (9) depends on the estimation method used to estimate the PopPK parameters, for example, the variance estimator when the restricted maximum estimation likelihood (REML) is used is shown in the following equation:

(10) $$\begin{array}{c} {\hat{\sigma }}_{i}^{2}=\frac{{\sum^{}}_{i=1}^{m}y_{i}-{\hat{y}}_{{\text{REML}}_{i}}}{n-p}. \end{array}$$

Equation (10) takes into account the degrees of freedom by subtracting the total of parameters *p* used in the estimation process [14] where ${\hat{y}}_{{\text{REML}}_{i}}$ is a vector of predicted values applying REML.

## 3. Implementation of the Software Robot

### 3.1. The Software Robot

**Figure 2:**
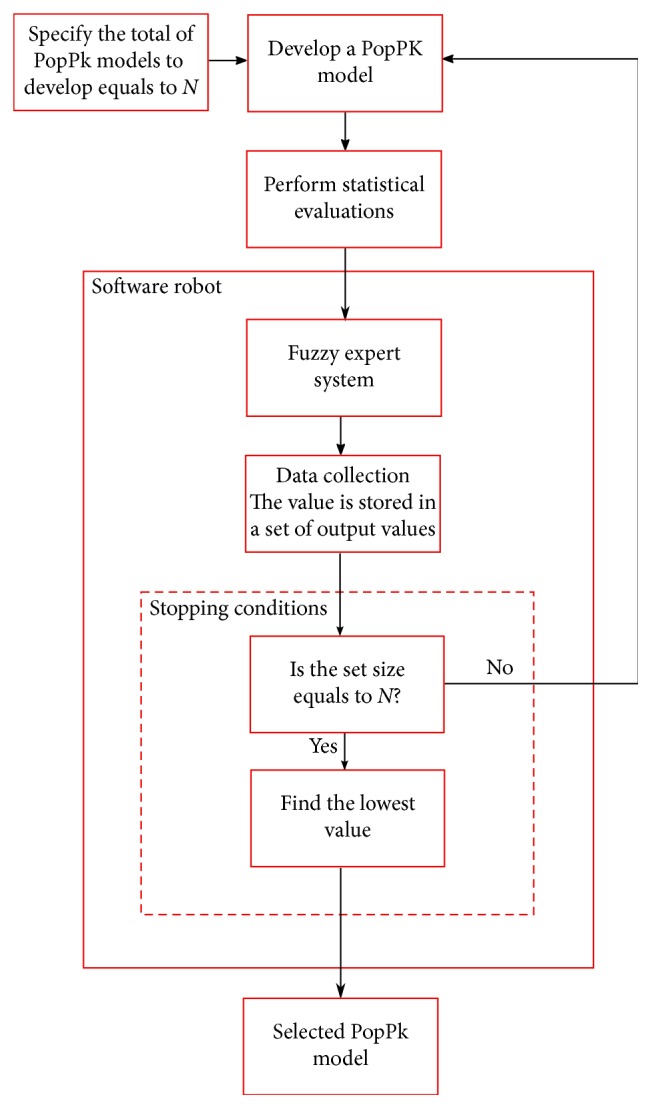
Block diagram.

The software robot contained a fuzzy expert system that takes the advantage of the human knowledge and their expertise in a given field to create linguistic descriptors for variables and create fuzzy sets that allow to control the behavior of phenomena under study or even as a tool for decision making [21]. In this work, we developed a software robot as shown in Figure 2 to evaluate indistinct versions of a PopPK model for an aminoglycoside antibiotic named tobramycin which has been the subject of many studies due to its narrow therapeutic window [22]. In this figure, first, we defined the number *N* of PopPK models to be developed taking into account the number of parameters and covariates. After that, we proceed to establish one by one the *N* PopPK models. Three statistical evaluation criteria are performed on each model, and they are AIC, SC, and MSE. The software robot uses them as the inputs for the fuzzy expert system, and it collects each defuzzified output into an array until all the *N* models have been evaluated. Then, the software robot chooses the best PopPK model which is the one that had the lowest value in the fuzzy evaluation, and this will be selected as the best PopPK model.

Thus, the evaluation made by our software robot will guide us to decide if a PopPK model of tobramycin is the best within a group of PopPK models of Tobramycin.

The variables are the evaluation criteria AIC, SC, and MSE presented in Equations (7), (8), and (10), respectively, and commonly used to evaluate a PopPK model, and their linguistic descriptors are the ranges of values. For example, the variable AIC has a range of values from 670 to 672.5 considered “Very Low”, from 670 to 675 considered “Low,” from 672.5 to 677.5 considered “Medium,” from 675 to 680 considered “High,” and from 677.5 to 680 considered “Very High.” “Very Low,” “Low,” “Medium,” “High,” and “Very High” are the linguistic descriptors for the sets of values for the variable AIC.

As is typical of the fuzzy theory, the sets of values overlap, and in this way, a value may partially belong to a set and have a degree of membership 0 ≤ *μ* ≤ 1 that is any place between zero and one, where *μ* represents the degree of membership. Thus, the value belongs to several sets with the total membership adding to one. Going back to the above example, the linguistic descriptors of “Very Low” and “Low” are two fuzzy sets for the variable AIC that may overlap, so an AIC of 670.5 could be mostly “Very Low” with *μ*=0.8 and somewhat “Low” with *μ*=0.2.

Implementing fuzzy sets with linguistic descriptors to perform a generalization of the output evaluation criteria, results of the fitted PopPK model, we may establish relationships between variables to evaluate the PopPK model by extracting fuzzy rules in terms used by a human expert of the form *IF* − *THEN*. In other words, a fuzzy system can perform a description of the phenomena under study, based on the antecedents and consequents presented in a fuzzy rule, that is in the form *IF ***condition***THEN ***action***rules* [23].

Using the three evaluation criteria AIC, SC, and MSE, we can set a knowledge base in such way that *IF* AIC is mostly “Very Low” *AND* CS is mostly “Very Low” *AND* MSE is also mostly “Very Low” *THEN* Evaluation is “OPTIMAL”. That is, under the fuzzy logic, we are not only going to have linguistic descriptors as the inputs of the fuzzy system but also output variables.

**Figure 3:**
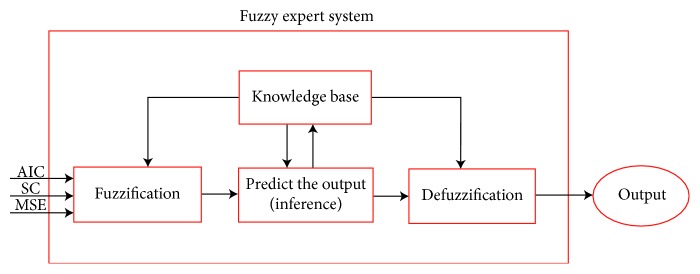
The crisp input values AIC, SC, and MSE are transformed into fuzzy inputs by the fuzzification unit which assigns the degree of membership to the fuzzy sets defined for the variables. The fuzzy system can then make inferences or predict outputs of the system by applying knowledge from the expert. Finally, the defuzzification unit transforms the obtained fuzzy output into a crisp value.

In summary, what a fuzzy system does is to transform crisp input values into fuzzy inputs through a fuzzification unit that establishes the degree of membership to the fuzzy sets for previously defined variables. Then, the fuzzy system uses a rulebase designed by the human expert to predict the fuzzy output of the phenomenon under study, whereby the fuzzy system has a defuzzification unit that transforms the fuzzy output into a crisp value. For this work, we created a fuzzy system with three crisp input variables as shown in Figure 3, the Mamdani max-min inferences to obtain the membership functions of the system and the centroid method to defuzzify the output.

### 3.2. Fuzzification

As we mentioned, the values of the three evaluation criteria for a developed PopPK model are used to perform a comparison between models, so we can finally decide the PopPK model that is “better,” which leads the researchers to deal with an MCDM problem. As an example, a researcher can get to the point in which he needs to decide between two models that have the best evaluation criteria values (Table 1).

In the example of Table 1, the model 2 has a better result in the AIC than model 1 for 1.866 units and in the MSE for 0.048 units. However, model 1 performs better in CS for a significant difference of almost 3 units (2.794). This type of ambiguity in the statistical results and the pressure to get the optimal PopPK model can result in a misinterpretation of the evaluation criteria to select the optimal PopPK model.

In this work, the values of the linguistic variables AIC, CS, and MSE calculated from the development of indistinct versions of a PopPK model of tobramycin were used as our fuzzy input sets. Their maximum and their minimum values are shown in Table 2.

The membership function for the three input variables is tagged as “Very Low,” “Low,” “Normal,” “Medium,” “High,” and “Very High,” and their corresponding parameter values are shown in Table 3.

The membership functions for the output variable “Evaluation” are represented by five membership functions, and the corresponding types and parameter values are shown in Table 4.

### 3.3. Fuzzy Rulebase

We created the fuzzy rulebase using the human knowledge of an expert who judged “how much more significant one attribute is than the other.” The inference is made on the rules *IF* − *THEN*; one rule for each fuzzy output set. The total of rules stems from the heuristic *R*=*l*^*n*^, where *R* is equal to the total of rules, *l* is equal to the number of linguistic descriptors, and *n* represents the number of input variables; thus in our case with *l*=5 and *n*=3 results in a total of 125 rules (Table 5).

#### 3.3.1. Defuzzifier

The fuzzified functions obtained from fuzzy inference are converted into numeric values, for example:

  IF the **AIC** is Low (677)
  AND **CS** is Low (686)
  AND **MSE** is High (.12)
  THEN the PopPK model is Low Acceptable **(75.2)**

The above is achieved by applying the method of center of gravity defuzzifier for *N* rules using the formula:

(11) $$\begin{array}{c} C=\frac{{\sum^{}}_{i=1}^{N}b_{i}\int^{}\mu \left(i\right)}{{\sum^{}}_{i=1}^{N}\int^{}\mu \left(i\right)}, \end{array}$$

where *C* represents the crisp output value, *b*_*i*_ represents the center of the membership function of the consequent of the rule *i*, and ∫*μ*(*i*) is the area under the membership function ∫*μ*(*i*) of the consequent in the rule *i*. In other words, the center of gravity method calculates the center of mass from the output membership functions.

**Figure 4:**
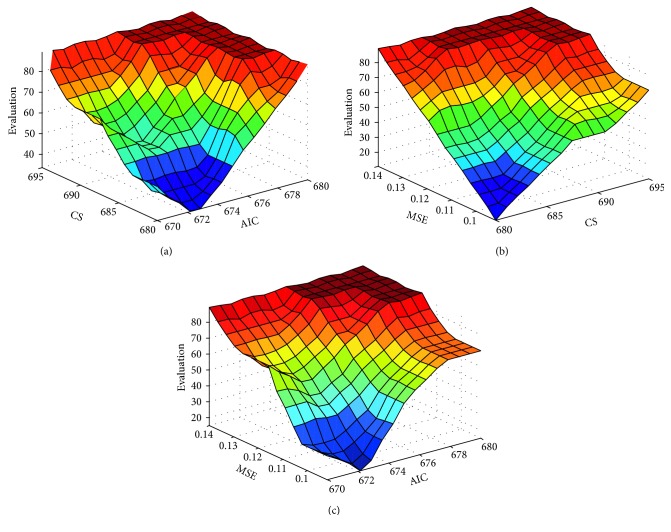
The figure shows the visualization of the system surface when the inputs AIC, CS, and MSE are interacting with each other. (a) The inputs AIC and CS are interacting. (b) The inputs MSE and CS are interacting. (c) The inputs MSE and AIC are interacting.

Figure 4 shows the global behavior of the fuzzy system for the relation between the input variables CS and AIC, MSE and CS, and MSE and AIC considering all their rules. For example, if the value of CS is “Very Low,” that is 681 and AIC is “High,” that is 678.5, then the PopPK model obtains a bad evaluation, or if the value of MSE is “Low,” that is 0.1 and the value of CS is also “Low”, that is 684, the model obtains an excellent evaluation. Another example will be a scenario where the value of MSE is “Medium,” that is 0.12, and the value of AIC is “Low,” that is 674.5, and the model obtains a good evaluation.

If it was required, the search for the lowest value could be done according to Algorithm 1. The crisp output value can be stored in an array together with other output values taken from other PopPK models that were evaluated previously and then compare them in an automated way to find the lowest value which will determine the optimal PopPK model.

## 4. Experimental Results for the Evaluation of Several PopPK Models of Tobramycin

This section describes the experiments performed to evaluate and compare 21 PopPK models based on the tobramycin database. We developed the PopPK Model of tobramycin in Matlab applying the single compartment model, as it is shown in Figure 1(a). The covariate model for both *V* and Cl were defined as linear.

The estimation of the parameters for all the generated PopPK models was made by applying REML. The optimization process was conducted with the quasi-Newton algorithm using the initial set values for fixed effects of 0.01 for Cl and 0.01 for *V* and with a maximum of 100 iterations. The same residual error model was applied to all the experiments. The only variations in the developed PopPK models are the covariate type and the number of parameters included in each model.

### 4.1. Experiment 1

After having evaluated 21 PopPK models using the evaluations criteria (7), (8), and (10), we end up with the 8 best PopPK models shown in Table 6.

Once we performed a simple analysis of the 8 PopPK models, we can easily decide that the PopPK models 1 and 2 have the worst values. However, considering the rest of the six PopPK models, it is hard to determine which model is the best given that some of their evaluations values are countered.

### 4.2. Experiment 2

In this experiment, we used the results of the evaluation criteria shown in Table 6 of the PopPK models 2, 4, 5, 6, 7, and 8, as input variables in our software robot as described in Section 3. The results of the evaluations made for these PopPK models are shown in Table 7, where now the fuzzy system evaluation (FSE) criteria results are presented for the six selected PopPK models.

## 5. Analysis of Results

The results of experiment 1 of Table 6 represent an example of the type of problem the researcher can face when working in the development of any PopPK model. In this particular case, Table 6 shows that the selection of the best PopPK model is not a trivial decision due to the time consumed to determine which PopPK model obtained the most significant evaluations in comparative with the rest of the models. For example, in Table 6, it can be seen that PopPK model 5 has best AIC evaluation than the PopPK models 4, 6, and 7, but worst evaluation in CS as well as worst evaluation in MSE than the PopPK model 6. The results of experiment 2 of Table 7 show that the fuzzy system gives the worst FSE value to PopPK model 7 (88.9). The PopPK model 7 has the worst evaluation of AIC, CS, and MSE than the PopPK model 4, which is the model with the best FSE value (31.6). It can be seen that the fuzzy system considered the trade-offs among the rest of standard statistical evaluation criteria: AIC, CS, and MSE provided a clearer picture of the order of the worst PopPK model to the best PopPK model.

## 6. Conclusions

Our results bolster the hypothesis that the software robot can be successfully implemented to evaluate PopPK models ensuring the selection of the best PopPK model when the choice of this becomes an MCDC problem. Given that the FSE criteria is built taking into account the classical evaluation criteria (AIC, CS, and MSE), we are able to only use the FSE as our unique automated evaluation criteria. This reduced the time and the error in the selection of the best PopPK model.

Another advantage is that if we want to use the same fuzzy system methodology for the evaluation of a different case study, for example, a PopPK model that involves another type of drug as phenobarbital, or the amount of individuals in the study is different, we only need to evaluate a certain amount of models to perform adjustments in the membership function ranges.

Our software robot performs fuzzy evaluations offering a stronger alternative to increase the efficiency in the selection of the best PopPK from a set. It can help the pharmaceutical scientist or the expert researchers in the area of computer intelligence to incorporate, expand, and improve the implementation of this software either in new versions of commercial software or in the development of new software to incorporate human expertise.

The proposed software robot can become a strong support to further studies regarding this novel approach by helping the pharmaceutical scientist or the expert researchers in the area of computer intelligence to incorporate, expand, and improve this development.

## Figures and Tables

**Algorithm 1:**
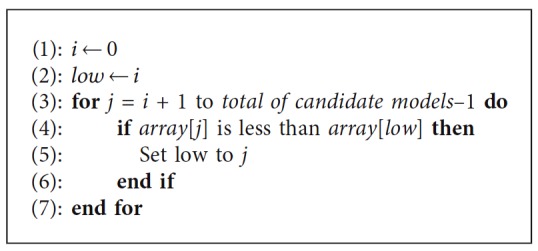
Find the lowest value algorithm.

**Table 1:** Example of the final evaluation results for a PopPK model.

PopPK model	AIC	CS	MSE
1	652.470	665.300	0.14
2	650.604	668.094	0.092

The best results are the values with red color.

**Table 2:** AIC, CS, and MSE values used for the fuzzy expert system.

Variable	Minimum value	Maximum value
AIC	670	680
CS	680	795
MSE	0.095	0.14

**Table 3:** Parameters of the membership function values for the input variables.

Linguistic variables	Linguistic term names, type shapes, and parameters					
	Very Low trapezoidal	Low triangular	Medium triangular	High triangular	Very High trapezoidal
AIC	[670 670 670.3 672.5]	[670 672.5 675]	[672.5 675 677.5]	[675 677.5 680]	[677.5 679.7 680 680]
CS	[676.6 679.6 680.4 683.4]	[680 683.8 687.5]	[683.8 687.5 691.3]	[687.5 691.3 695]	[691 695 695 698]
MSE	[0.092 0.092 0.09397 0.104]	[0.0921 0.104 0.116]	[0.104 0.116 0.128]	[0.116 0.128 0.14]	[0.128 0.1384 0.14 0.14]

**Table 4:** Parameters of the membership function values for the output variable.

Linguistic variables	Lingustic term names, type shapes, and parameters					
	Optimal trapezoidal	High Acceptable	Acceptable triangular	Low Acceptable triangular	Rejected trapezoidal
Evaluation	[0 0 10 30]	[10 30 50]	[30 50 70]	[50 70 90]	[70 90 100 100]

**Table 5:** Fuzzy rules.

Rule number	Linguistic inputs		Linguistic output	
	AIC	CS	MSE	Evaluation
1	Very Low	Very Low	Very Low	Optimal
2	Very Low	Very Low	Low	High Acceptable
3	Very Low	Very Low	Medium	Acceptable
4	Very Low	Very Low	High	Low Acceptable
5	Very Low	Very Low	Very	Optimal
7	Very Low	Low	Low	High Acceptable
8	Very Low	Low	Medium	Acceptable
9	Very Low	Low	High	Low Acceptable
10	Very Low	Low	Very High	Rejected
11	Very Low	Medium	Very Low	High Acceptable
12	Very Low	Medium	Low	High Acceptable
13	Very Low	Medium	Medium	Low Acceptable
14	Very Low	Medium	High	Low Acceptable
15	Very Low	Medium	Very High	Rejected
16	Very Low	High	Very Low	Acceptable
17	Very Low	High	Low	Acceptable
18	Very Low	High	Medium	Low Acceptable
19	Very Low	High	High	Rejected
20	Very Low	High	Very High	Rejected
21	Very Low	Very High	Very Low	Low Acceptable
22	Very Low	Very High	Low	Low Acceptable
23	Very Low	Very High	Medium	Rejected
24	Very Low	Very High	High	Rejected
25	Very Low	Very High	Very High	Rejected
26	Low	Very Low	Very Low	Optimal
27	Low	Very Low	Low	Optimal
28	Low	Very Low	Medium	High Acceptable
29	Low	Very Low	High	Low Acceptable
30	Low	Very Low	Very High	Rejected
31	Low	Low	Very Low	Optimal
32	Low	Low	Low	High Acceptable
33	Low	Low	Medium	Acceptable
34	Low	Low	High	Low Acceptable
35	Low	Low	Very High	Rejected
36	Low	Medium	Very Low	Optimal
37	Low	Medium	Low	High Acceptable
38	Low	Medium	Medium	Acceptable
39	Low	Medium	High	Low Acceptable
40	Low	Medium	Very High	Rejected
41	Low	High	Very Low	High Acceptable
42	Low	High	Low	Acceptable
43	Low	High	Medium	Low Acceptable
44	Low	High	High	Rejected
45	Low	High	Very High	Rejected
46	Low	Very High	Very Low	Acceptable
47	Low	Very High	Low	Low Acceptable
48	Low	Very High	Medium	Rejected
49	Low	Very High	High	Rejected
50	Low	Very High	Very High	Rejected
51	Medium	Very Low	Very Low	Optimal
52	Medium	Very Low	Low	High Acceptable
53	Medium	Very Low	Medium	Acceptable
54	Medium	Very Low	High	Low Acceptable
55	Medium	Very Low	Very High	Rejected
56	Medium	Low	Very Low	High Acceptable
57	Medium	Low	Low	High Acceptable
58	Medium	Low	Medium	Acceptable
59	Medium	Low	High	Rejected
60	Medium	Low	Very High	Rejected
61	Medium	Medium	Very Low	Acceptable
62	Medium	Medium	Low	Acceptable
63	Medium	Medium	Medium	Low Acceptable
64	Medium	Medium	High	Rejected
65	Medium	Medium	Very High	Rejected
66	Medium	High	Very Low	Acceptable
67	Medium	High	Low	Low Acceptable
68	Medium	High	Medium	Rejected
69	Medium	High	High	Rejected
70	Medium	High	Very High	Rejected
71	Medium	Very High	Very Low	Low Acceptable
72	Medium	Very High	Low	Low Acceptable
73	Medium	Very High	Medium	Rejected
74	Medium	Very High	High	Rejected
75	Medium	Very High	Very High	Rejected
76	High	Very Low	Very Low	Acceptable
77	High	Very Low	Low	Acceptable
78	High	Very Low	Medium	Low Acceptable
79	High	Very Low	High	Rejected
80	High	Very High	Very High	Rejected
81	High	Low	Very Low	Low Acceptable
82	High	Low	Low	Low Acceptable
83	High	Low	Medium	Rejected
84	High	Low	High	Rejected
85	High	Low	Very High	Rejected
86	High	Medium	Very Low	Low Acceptable
87	High	Medium	Low	Low Acceptable
88	High	Medium	Medium	Rejected
89	High	Medium	High	Rejected
90	High	Medium	Very High	Rejected
91	High	High	Very Low	Low Acceptable
92	High	High	Low	Rejected
93	High	High	Medium	Rejected
94	High	High	High	Rejected
95	High	High	Very High	Rejected
96	High	Very High	Very Low	Low Acceptable
97	High	Very High	Low	Low Acceptable
98	High	Very High	Medium	Rejected
99	High	Very High	High	Rejected
100	High	Very High	Very High	Rejected
101	Very High	Very Low	Very Low	Acceptable
102	Very High	Very Low	Low	Low Acceptable
103	Very High	Very Low	Medium	Rejected
104	Very High	Very Low	High	Rejected
105	Very High	Very Low	Very High	Rejected
106	Very High	Low	Very Low	Low Acceptable
107	Very High	Low	Low	Low Acceptable
108	Very High	Low	Medium	Rejected
109	Very High	Low	High	Rejected
110	Very High	Low	Very High	Rejected
111	Very High	Medium	Very Low	Low Acceptable
112	Very High	Medium	Low	Low Acceptable
113	Very High	Medium	Medium	Rejected
114	Very High	Medium	High	Rejected
115	Very High	Medium	Very High	Rejected
116	Very High	High	Very Low	Rejected
117	Very High	High	Low	Rejected
118	Very High	High	Medium	Rejected
119	Very High	High	High	Rejected
120	Very High	High	Very High	Rejected
121	Very High	Very High	Very Low	Rejected
122	Very High	Very High	Low	Rejected
123	Very High	Very High	Medium	Rejected
124	Very High	Very High	High	Rejected
125	Very High	Very High	Very High	Rejected

**Table 6:** Results of the 8 best PopPK model of tobramicyn. The covariates used are weight (WT), age, height (HT), and body mass index (BMI).

PopPK	# of estimated				
Model	Parameters	Covariates	AIC	CS	MSE
1	6	(AGE/*V*)/(WTCl)	682.04	698.2	0.0936
2	6	(SEX,AGE/*V*)	679.9	695	0.093
3	7	(WT,AGE/*V*)/(WT/Cl)	683.6	701.1	0.093
4	6	(SEX,AGE/*V*)	673.1	689.2	0.09336
5	9	(SEX,AGE/*V*)/(SEX,AGE,WT/Cl)	670.4	693	0.0936
6	7	(SEX, AGE/*V*) (SEX/Cl)	672.7	691	0.093
7	7	(WT, AGE, SEX/Cl)	675.3	691.3	0.139
8	10	(WT, HT/*V*)/(WT, AGE, HT, BMI/Cl)	682.7	707.5	0.0928

**Table 7:** Summary of all evaluation criteria applied. Model 4 is the best evaluated by the fuzzy system.

PopPK	# of estimated					
Model	Parameters	Covariates	AIC	CS	MSE	FSE
2	6	(WT,AGE/*V*)	679.9	695	0.093	88.4
4	6	(SEX,AGE/*V*)	673.1	689.2	0.09336	31.6
5	9	(SEX,AGE/*V*)/(SEX,AGE,WT/Cl)	670.4	693	0.0936	55.6
6	7	(SEX, AGE/*V*) (SEX/Cl)	672.7	691	0.093	34.4
7	7	(WT, AGE, SEX/Cl)	675.3	691.3	0.139	88.9
8	10	(WT, HT/*V*)/(WT, AGE, HT, BMI/Cl)	682.7	707.5	0.0928	88.7

## Data Availability

The data of our document are available upon request to any of the authors.
